# Examining health-related quality of life in pediatric cancer patients with febrile neutropenia: Factors predicting poor recovery in children and their parents

**DOI:** 10.1016/j.eclinm.2021.101095

**Published:** 2021-08-20

**Authors:** Anna Crothers, Gabrielle M Haeusler, Monica A Slavin, Franz E Babl, Francoise Mechinaud, Robert Phillips, Heather Tapp, Bhavna Padhye, David Zeigler, Julia Clark, Thomas Walwyn, Leanne Super, Frank Alvaro, Karin Thursky, Richard De Abreu Lourenco

**Affiliations:** a Centre for Health Economics Research and Evaluation, University of Technology Sydney, Australia; b Department of Infectious Diseases, Peter MacCallum Cancer Centre, Melbourne, Victoria, Australia; c NHMRC National Centre for Infections in Cancer, Sir Peter MacCallum Department of Oncology, University of Melbourne, Parkville, Australia; d The Paediatric Integrated Cancer Service, Parkville, Australia; e Infection Diseases Unit, Department of General Medicine, Royal Children's Hospital, Parkville, Australia; f Murdoch Children's Research Institute, Parkville, Australia; g Department of Paediatrics, University of Melbourne, Parkville, Australia; h Department of Medicine, University of Melbourne, Parkville, Victoria, Australia; i NHMRC National Centre for Antimicrobial Stewardship, The Peter Doherty Institute for Infection and Immunity, Melbourne, Australia; j Victorian Infectious Diseases Service, The Peter Doherty Institute for Infection and Immunity, Melbourne, Australia; k Department of Emergency Medicine, Royal Children's Hospital, Parkville, Australia; l Unité d'hématologie immunologie pédiatrique, Hopital Robert Debré, APHP Nord Université de Paris, France; m Centre for Reviews and Dissemination, University of York, York, UK; n Leeds Children's Hospital, Leeds General Infirmary, Leeds, UK; o Department of Oncology, Women's and Children's Hospital, Adelaide, Australia; p Cancer Centre for Children, Westmead Children's Hospital, Sydney, Australia; q Kid's Cancer Centre, Sydney Children's Hospital, Sydney, Australia; r Infection Management Service, Queensland Children's Hospital, Children's Health Queensland Hospital and Health Service, Brisbane, Australia; s Department of Oncology, Perth Children's Hospital, Perth, Australia; t Children's Cancer Centre, Monash Children's Hospital, Monash Health, Melbourne, Australia; u Children's Cancer Department, John Hunter Children's Hospital, University of Newcastle, Newcastle, Australia

**Keywords:** Cancer, Child, Febrile neutropenia, Fever, Health-related quality of life, Parent, pediatric cancer, Pediatric oncology

## Abstract

**Background:**

The impact febrile neutropenia (FN) has on the health-related quality of life (HRQoL) of children with cancer and their families is poorly understood. We sought to characterize the course of child and parent HRQoL during and following FN episodes.

**Method:**

Data on HRQoL were collected in the multisite Australian Predicting Infectious ComplicatioNs in Children with Cancer (PICNICC) study. Participants were enrolled between November 2016 to January 2018. The Child Health Utility (CHU9D) was used to assess HRQoL in children (*N* = 167 FN events) and the Assessment of Quality of Life (AQoL-8D) was used to assess HRQoL parents (*N* = 218 FN events) at three time points: 0–3 days, 7-days and 30-days following the onset of FN. Group-based trajectory modeling (GBTM) was used to characterize the course of HRQoL.

**Findings:**

For children, three distinct groups were identified: persistently low HRQoL over the 30-day course of follow-up (chronic: *N* = 78/167; 47%), increasing HRQoL after the onset of FN to 30 days follow-up (recovering: *N* = 36/167; 22%), and persistently high HRQoL at all three timepoints (resilient: *N* = 53/167; 32%). Applying these definitions, parents were classified into two distinct groups: chronic (*N* = 107/218, 49%) and resilient (*N* = 111/218, 51%). The child being male, having solid cancer, the presence of financial stress, and relationship difficulties between the parent and child were significant predictors of chronic group membership for both parents and children. Children classified as high-risk FN were significantly more likely to belong to the recovery group. Being female, having blood cancers and the absence of financial or relationship difficulties were predictive of both parents and children being in the resilient group.

**Interpretation:**

Approximately half the children and parents had chronically low HRQoL scores, which did not improve following resolution of the FN episode. The child's sex, cancer type, and presence of financial and relationship stress were predictive of chronic group membership for both parents and children. These families may benefit from increased financial and psychosocial support during anti-cancer treatment.

**Funding:**

National Health and Medical Research Council Grant (APP1104527).

**Research in context**

**Evidence before this study**

We searched PubMed and MEDLINE in December 2020, for studies published in English, using the search terms “febrile neutropenia”, “HRQoL, “Quality of Life”, “child”, “pediatric” and “cancer”.

There is very limited published data on both child and parent HRQoL throughout the entire FN episode and to date, predictors of poor HRQoL in these populations have not been characterised. Prior research has examined health-related quality of life (HRQoL) using non-standardised measures, which do not allow for comparison across studies or benchmarking. Further, HRQoL was only assessed during the FN episode but no studies examined HRQoL following the resolution of an FN episode.

**Added value of this study**

As part of the prospective Australian Predicting Infectious ComplicatioNs in Children with Cancer (PICNICC) study, we detailed the course of HRQoL for children and their parents at the onset of FN (0–1 days after FN onset), after its resolution at 7-days and at 30-days from FN. This is the first study of its kind and the first time the Child Health Utility (CHU9D) tool has been used in a pediatric cancer population. To our knowledge, it is also the first study to apply group-based trajectory modeling to patients with reoccurring events and multiple courses of follow-up, allowing us to examine changes in trajectory group membership over time.

**Implication of all the available evidence**

We show that the onset of FN has a significant and heterogeneous impact on the HRQoL of children with cancer and their parents, being classified into three groups: chronic (persistently low HRQoL at FN-onset and throughout follow-up); recovery (HRQoL was initially low at FN-onset but recovered during follow-up); and resilient (persistently high HRQoL at both FN-onset and throughout follow-up) trajectory groups. Risk factors for chronic group membership included the child being male, having solid cancer, undergoing intensive chemotherapy, financial stress and the presence of relationship difficulties between parent and child. These factors could be used to identify children and families who would benefit from additional financial and psychological support.

Alt-text: Unlabelled box

## 1 Introduction

In children with cancer, febrile neutropenia (FN) is a common and disruptive complication of anti-cancer treatment, occurring at a rate of 0.75 episodes per 30 days of neutropenia and 0.15 per month of chemotherapy exposure [1,2]. While a bacterial infection is documented in around 20% of children with FN and 3% require intensive-care-unit (ICU) level care, contemporary studies still report that over 50% of children recover quickly and do not have a clinical infection [3]. A risk-stratified approach to care should be considered in FN, enabling low-risk patients to be managed with reduced-intensity treatment, including oral antibiotics, with the option for administration in the comfort of their own home [3].

Despite the frequency with which FN occurs in children with cancer, very few studies have quantified the health-related quality of life (HRQoL) of children and their parents during these episodes [4], [5], [6]. To our knowledge, the only study to assess HRQoL in children and their parents during an FN episode was Orme et al. 2014 [5]. However, this study assessed HRQoL using the visual analogue scale which has limited external comparability. Further, this study only assessed HRQoL during the FN episode but did not examine HRQoL after the FN episode resolved.

Understanding and characterizing the course of children and their family's HRQoL following the onset of FN, and identifying factors predicting poor outcomes may enable clinicians to optimize treatment algorithms that maximize HRQoL and patient experience [2]. Moreover, characterizing and quantifying the HRQoL effects associated with FN is necessary to assess the cost effectiveness of FN care. This facilitates the assessment of the cost-effectiveness of existing or emerging approaches to the management of FN (in particular for low-risk FN) [7].

The Australian Predicting Infectious ComplicatioNs in Children with Cancer (PICNICC) study was a multi-site, prospective validation study that also captured comprehensive HRQoL and economic data on children with cancer and FN. Our study used HRQoL data from PICNICC to describe the course of HRQoL of children and their parents during and following an FN episode. We hypothesised that FN would have a temporary deleterious and heterogenous impact on HRQoL, which would dissipate following FN resolution. Therefore, this study sought to describe individual variation between children and their parents and to identify predictors of poor HRQoL.

## 2 Methods

### 2.1 Participants and procedures

The Australian PICNICC study is a prospective, multicentre, observational study (Australian New Zealand Clinical Trials Registry 12616001440415) conducted in eight Australian tertiary pediatric hospitals, which was open to recruitment from November 2016 to January 2018. Detailed methodology is described elsewhere  [3]. Briefly, children with cancer on active treatment who were admitted to hospital or presented to emergency, outpatient or day-chemotherapy departments with fever or clinical instability were eligible for inclusion. Fever was defined as a temperature ≥38 °C and neutropenia was defined as an absolute neutrophil count (ANC) <1000 cells/mm^3^. Children with a hematopoietic stem cell transplant (HSCT) within three months were excluded. Multiple, discrete FN episodes per patient were allowed [8].

During the study period, children were managed according to hospital FN guidelines. Risk stratification or home-based treatment of low risk FN was not routinely used.

### 2.2 Ethics

This study had primary approval from the Royal Children's Hospital Melbourne Human Research Ethics Committee (RCH HREC 36040A) and was ratified by the University of Technology Sydney HREC ETH17–1128. Informed parent consent was obtained prior to the child's enrolment in the study.

### 2.3 Main outcome measure and HRQoL instruments

As part of the Australian PICNICC study, both children and parents were invited to complete validated HRQoL instruments during the FN episode. Children's HRQoL was assessed using the Children's Health Utility Index 9 Dimension (CHU9D), a child-specific multi-attribute utility instrument (MAUI) that assesses HRQoL over the domains of worry, sadness, pain, tiredness, annoyance, school, sleep, daily routine and activities. The CHU9D is validated for use in children aged seven to seventeen years, with proxy completion by the parent for children age two to six years [9]. Parent's HRQoL was assessed using the Assessment of Quality of Life 8 Dimensions (AQoL-8D), a generic MAUI, which assesses HRQoL over the two super dimensions ‘physical health’ (independent living, senses, pain) and ‘mental health’ (self-worth, coping, relationships, happiness) [10].

HRQoL data were collected at three time points following FN onset: within 0–3 days (in hospital) and at 7-days (at discharge) and at 30-days. Children were included if they had completed the CHU9D questionnaire for at least one FN episode. Parents were included if they had completed the AQoL-8D questionnaire for at least of one of their child's FN episodes. A complete case analysis was used.

Both questionnaires allow their HRQoL scores to be expressed as utility values on a scale from <0 to 1 (1 indicating perfect health and <0 depicting states worse than death) [10]. Australian-specific utility values were applied to the completed questionnaires to measure HRQoL according to the protocols specified by the developers [9,10].

### 2.4 Statistical analysis

Group-based trajectory modeling (GBTM) was used to identify distinct HRQoL trajectories over time within the FN episode. This method does not require participants to have complete data across all time points and allows for categorization of heterogeneous populations [11]. The GBTM is robust to serial correlation when applied to patients with multiple FN episodes and multiple courses of follow-up as it assumes conditional independence. That is for each individual within a given trajectory group, the distribution of the current observations of the outcome variable (y_t_) is independent of prior values (y_t-1_)  [12,13].

The GBTM uses maximum likelihood to model the probability of a specified number of underlying latent trajectories and their shape. Statistical criteria for ascertaining the best fitting model included Bayesian Information Criterion (BIC). Group-specific average posterior probabilities and estimated group sizes were also used to calculate group-specific odds of correct classification to assess entropy. Other criteria included nonoverlapping confidence intervals (CIs), distinct average posterior probabilities across groups, and whether the additional trajectory added clinically relevant information (see Table S8 and Table S9).

Statistical analyses were performed using STATA/SE 16 and the *traj* Plugin for STATA (StataCorp, College Station, TX). A censored normal distribution (minimum = −0.10, maximum =1) was used in the model and applied to HRQoL values for both parent and child. Univariate associations between predictors and HRQoL trajectories were examined using trajectory risk factor analyses. In addition, a multivariate model including all predictive factors was investigated. All variables found to be significant in univariate analyses at p-value ≤0.05 were included in subsequent multivariate analyses. Age, sex, and time with cancer were included in multivariate analyses regardless of significance as they were considered important control variables. To account for multiple FN episodes per patient, multinomial and logistic regression with cluster robust standard error were used (see Table S7). A subsequent exploratory analysis examined the linear association between these risk factors and parent and child HRQoL using mixed-effects linear regression (see Table S6).

### 2.5 Risk factors

Child's age, sex, hospital length of stay (LOS), antibiotic treatment duration, FN episode duration, FN risk status (according to the PICNICC rule recalibrated in the Australian population) [14], time to first antibiotic dose after hospital presentation, chemotherapy intensity (more or less intensive than acute lymphoblastic leukemia (ALL) maintenance), and cancer diagnosis were reported as part of the PICNICC variables. Cancer diagnosis was dichotomously characterised as “blood cancer” (i.e. lymphoma/leukemia) or “solid cancer” (i.e. sarcoma, brain tumours or other solid tumours). The presence of financial stress and relationship distress between the parent and child was assessed using two items from the Care-related Quality of Life instrument (CarerQol) questionnaire [15]: (1) I have financial problems due to my child's cancer and (2) I have relationship problems with my child. Responses were dichotomised as “no” and “some/a lot” (herein referred to as relationship strain).

### 2.6 Role of funding source

This study was funded by National Health and Medical Research Council (NHMRC) Project Grant (APP1104527). The NHMRC was not involved in study design, data collection, analysis or manuscript preparation or approval. The corresponding author (Anna Crothers), and her supervisor (Richard De Abreu Lourenco) had full access to all the data in the study and had final responsibility for the decision to submit for publication.

## 3 Results

### 3.1 Sample characteristics

Of the 462 children enrolled in the Australian PICNICC study, 117 (25%) children had completed the CHU9D survey at least once, for at least one FN episode and 133 (29%) of children had at least one parent that had completed the AQoL-8D survey at least once, for at least one of the child's FN episodes (Table 1). Of the participants included in this analysis with available HRQoL data, 51% (*N* = 119/234) were female, mean age was 7.8 years (SD = 4.8) and approximately 80% (*N* = 188/234) were classified, using the PICNICC FN rule as ‘high-risk’ [14]. The average number of FN episodes reported per child was 1.8 (SD = 1.0), with 55% (*N* = 129/234) of children experiencing two or more FN episodes during the study period. Of the parents who completed the AQoL-8D survey, most were female (*N* = 71/80; 89%) and most were university educated (*N* = 44/80; 55%) (see Table 1).

**Table 1:** Sample characteristics (reported per an FN episode, unless otherwise specified).

Mean (standard deviation), unless otherwise specified	Parent or Child^a^	Parent	Child
Total number of FN episodes with available HRQoL data	234	218	167
Number of FN episodes per patient with available HRQoL data - One FN episode - Two FN episodes - Three FN episodes - ≥ Four FN episodes	105 (45%) 90 (39%) 18 (8%) 21 (9%)	99 (45%) 80 (37%) 18 (8%) 21 (10%)	77 (46%) 62 (37%) 24 (14%) 4 (2%)
Mean number of FN episodes reported per child with available HRQoL data	1.8 (1.0)	1.8 (1.0)	1.7 (0.8)
Mean number of days between FN episodes	25.9 (40.2)	26.3 (40.8)	27.8 (42.2)
FN treatment assignment - Hospital care - Home-based care (HITH)	209 (89%) 26 (11%)	192 (88%) 26 (12%)	149 (89%) 18 (11%)
Child's sex - Male, n (%) - Female, n (%)	115 (49%) 119 (51%)	108 (50%) 110 (50%)	83 (50%) 84 (50%)
Parent completing the HRQoL survey's sex b - Male, n (%) - Female, n (%)	9 (11%) 71 (89%)	6 (9%) 62 (91%)	8 (13%) 60 (87%)
Mean child's age at FN onset (years)	7.8 (4.8)	7.9 (4.9)	8.6 (4.3)
Cancer Type - Blood cancer (lymphoma/leukemia) - Solid cancer, n (%)	155 (66%) 79 (34%)	146 (67%) 72 (33%)	110 (66%) 57 (34%)
Mean length of hospital stay, days (SD)	8.5 (11.5)	8.7 (11.8)	8.0 (11.8)
Length of hospital stay (dichotomised at median) - 0–5 days, n (%) - > 5 days, n (%)	121 (52%) 113 (48%)	112 (51%) 106 (49%)	89 (53%) 78 (47%)
Chemotherapy intensity - Non-intensive (equivalent to ALL maintenance), n (%) - Intensive (more intensive than ALL maintenance), n (%)	35 (15%) 199 (85%)	32 (15%) 186 (85%)	31 (19%) 136 (82%)
Mean duration of antibiotic treatment, days (SD)	7.3 (7.8)	7.4 (7.3)	7.0 (7.4)
Duration of antibiotic treatment (dichotomised at median) - 0–4 days, n (%) - > 4 days, n (%)	104 (44%) 130 (56%)	97 (45%) 121 (56%)	78 (47%) 89 (53%)
Mean duration of FN episode, days (SD)	1.3 (2.1)	1.3 (2.0)	1.4 (2.1)
Mean time to first antibiotic after hospital presentation, hours (SD)	1.0 (0.8)	.0 (0.8)	1.0 (08)
Time to first antibiotic - Within 1 hour of hospital presentation, n (%) - > 1 hour after hour of presentation, n (%)	142 (60%) 92 (39%)	134 (62%) 74 (39%)	103 (62%) 64 (38%)
Mean time with cancer (months)	7.9 (9.9)	7.9 (10.0)	8.2 (10.3)
Time with cancer (dichotomised at median) - ≤ 4.5 months, n (%) - > 4.5 months, n (%)	113 (48%) 121 (52%)	106 (49%) 113 (51%)	77 (46%) 90 (54%)
PICNICC FN risk status^14^ - Low risk, n (%) - High risk, n (%)	46 (20%) 188 (80%)	45 (21%) 173 (79%)	36 (22%) 131 (78%)
CHU9D questionnaire completed by: - Parent proxy, n (%) - Child, n (%)	87 (53%) 78 (47%)		87 (53%) 78 (47%)
Mean CHU9D score at: - 0–3 days after FN onset - 7-days after FN onset - 30-days FN onset	0.39 (0.28) 0.48 (030) 0.51 (0.32)	0.38 (0.28) 0.48 (0.29) 052 (0.32)	0.39 (0.28) 0.48 (030) 0.51 (0.32)
Mean AQoL-8D score at: - 0–3 days after FN onset - 7-days after FN onset - 30-days FN onset	0.65 (0.19) 0.65 (0.19) 0.68 (0.20)	0.65 (0.19) 0.65 (0.19) 0.68 (0.20)	0.68 (0.19) 0.65 (0.19) 0.71 (0.20)
CarerQol relationship at FN onset - I have no relationship problems with my child - I have some of relationship problems with my child - I have a lot of relationship problems with my child	141 (65%) 69 (31%) 8 (4%)	141 (65%) 69 (31%) 8 (4%)	86 (58%) 53 (37%) 6 (4%)
CarerQol finance at FN onset - I have no financial problems due to my child's cancer - I have some of financial problems due to my child's cancer - I have a lot of financial problems due to my child's cancer	39 (18%) 129 (59%) 50 (23%)	39 (18%) 129 (59%) 50 (23%)	27 (17%) 91 (63%) 27 (19%)
Parent's highest education b -High school - Trade or certificate - University degree	11 (14%) 25 (31%) 44 (55%)	10 (15%) 19 (28%) 39 (57%)	6 (10%) 17 (28%) 37 (62%)

Abbreviations: AQoL-8D = Assessment of Quality of Life – 8 Dimensions, CarerQol = Care-related Quality of Life instrument, CHU9D = CHU9D – 9 Dimensions, FN = febrile neutropenia, HITH = hospital in the home; HRQoL = health-related quality of life, parent proxy = parent completed the CHU9D on the child's behalf; PICNICC = Predicting Infectious Complications in Children with Cancer.

Notes:

a Parent and/or the child had completed the HRQoL survey for the FN episode.

b Demographic information relating to the child's parents was collected retrospectively. Hence, most families did not complete the retrospective demographic questionnaire.

In accordance with local and state-based clinical guidelines, most children (60%) received antibiotics within one hour of hospital presentation [16]. Overall, the mean LOS was 8.7 (SD = 11.5) days and children typically received antibiotics for 7 days (mean = 7.3; SD = 7.8). Most children had blood cancers (*N* = 155/234; 70%) and were undergoing intensive chemotherapy at the onset of FN (*N* = 199/234; 85%). Most parents reported financial stress due to their child's cancer (*N* = 179/218; 80%) and one third reported relationship strain with their child (*N* = 77/218; 35%) (see Table 1).

FN episodes with available HRQoL information had a shorter hospital stay and a lower proportion were accounted for by solid cancers compared with FN episodes without HRQoL information (see Table S3). Otherwise, there were no significant differences between these groups.

### 3.2 HRQoL results and trajectories in children and parents

**Fig. 1:**
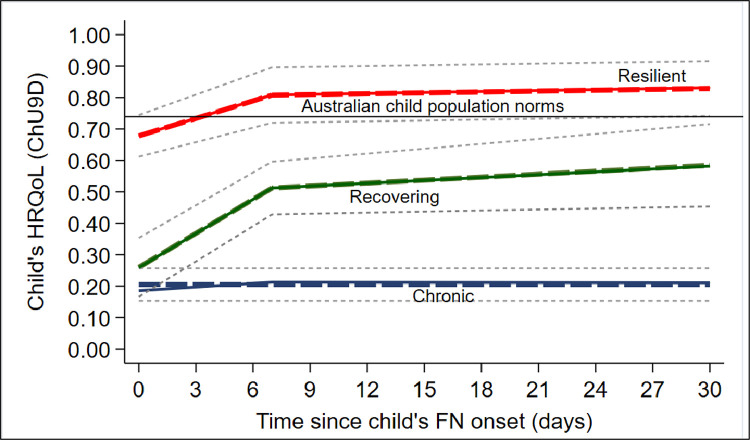
Predicted (dotted lines) and actual (solid lines) of child's HRQoL at 0–1, 7- and 30-days after the onset of the FN episode *Solid lines depict the actual average CHU9D scores of children in the ‘chronic’ (solid blue line; N* *=* *78/167, 47%), ‘recovering’ (sold green line, N* *=* *36/167, 22%) and ‘resilient’ (solid red line, N* *=* *53/167, 32%) groups at 0–3, 7 and 30 days after the onset of the FN episode. The solid black line depicts Australian population norms (mean = 74; SD = 0.15) [**9**]. The dotted blue, green and red lines depict the predicted trajectory of children's CHU9D scores in the ‘chronic’, ‘recovering’ and ‘resilient’ groups, respectively. The dotted gray lines depict the 95% confidence intervals of each trajectory group* Abbreviations: CHU9D = CHU9D – 9 Dimensions, FN = febrile neutropenia, HRQoL = health-related quality of life; SD = standard deviation.

Based on the children's HRQoL scores following FN, the best fitting trajectory model identified three patterns: chronic (*N* = 78/167; 47%), recovering (*N* = 36/167; 22%) and resilient (*N* = 53/167; 32%) (Fig. 1). Children in the chronic group had HRQoL scores that were relatively low, at both the onset of FN (mean = 0.19; 95% confidence interval (CI): 0.14, 0.23) and over the 30-day course of follow-up (mean = 0.21; 95% CI: 0.17, 0.26). Children in the recovery group had initially low HRQoL scores at the onset of FN (mean = 0.25; 95% CI: 0.19, 0.30), which improved after the resolution of the FN episode (typically 7-days after FN onset; mean = 0.54; 95% CI: 0.49, 0.60). Although HRQoL never approached Australian population norms (mean = 0.74; 95% CI: 0.72, 0.76) depicted by a solid black line in Fig. 1) [9]. Children in the resilient group had relatively high HRQoL scores, that were initially below population norms at the onset of FN but improved after the resolution of the FN episode and were comparable to Australian population norms [8]. Paired sample t-tests found that while children in the recovery (mean difference (MD) = 0.34, 95% CI: 0.27,0.41; p-value ≤ 0.001) and resilient (MD = 0.15, 95% CI: 0.07, 0.23; p-value ≤ 0.001) trajectory groups experienced significant improvements in HRQoL following the resolution of the FN episode (7-days after FN onset), children in the chronic group experienced no significant improvement in HRQoL over the 30-day course of follow-up (MD = −0.02, 95% CI: −0.07, 0.11; p-value > 5%) (see Table S4).

**Fig. 2:**
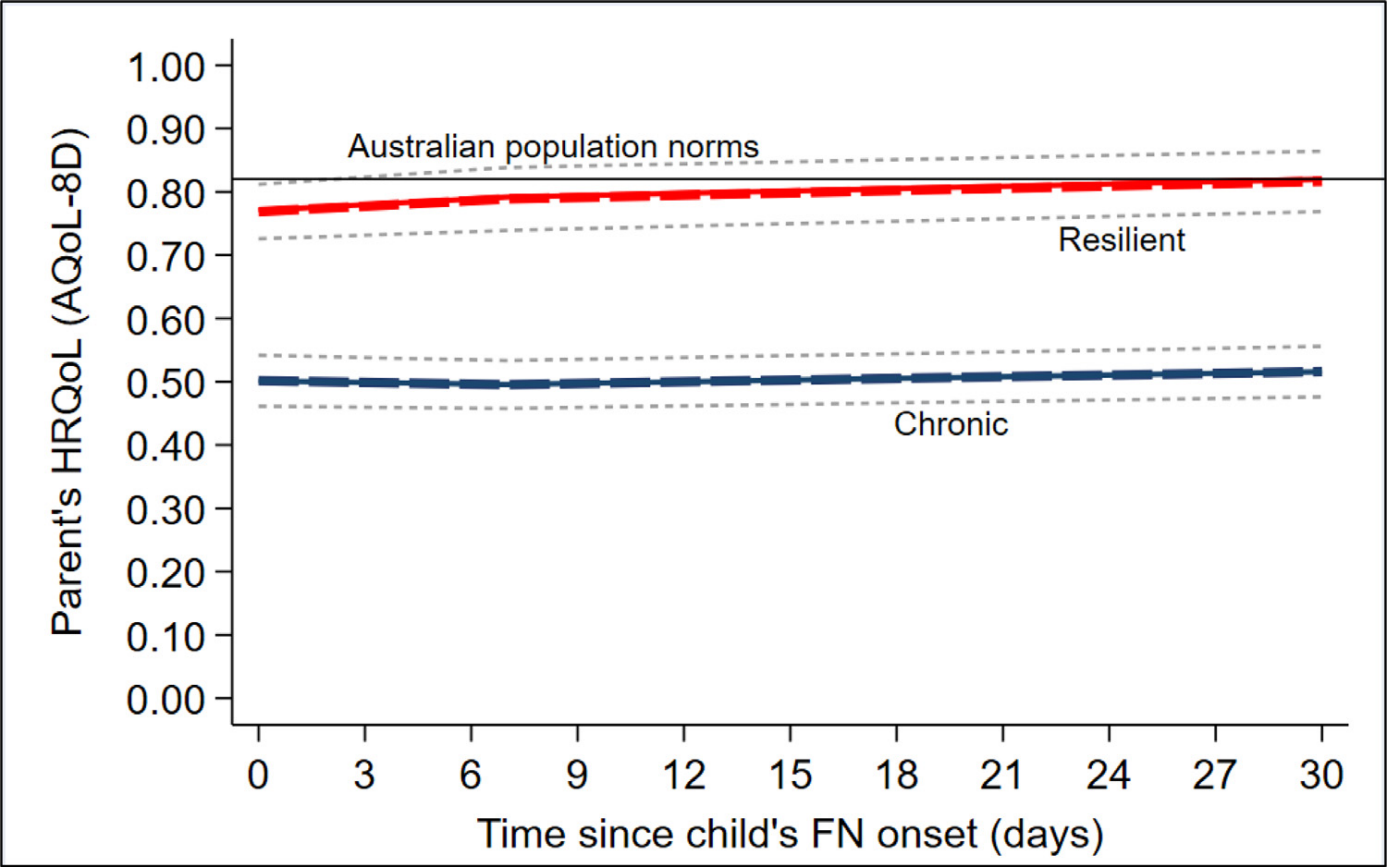
Predicted (dotted lines) and actual (solid lines) of parents HRQoL at 0–1, 7- and 30-days after the onset of the child's FN episode *Solid lines depict the actual average AQoL-8D scores of parents in the ‘chronic’ (solid blue line; N* *=* *107/218; 49%) and ‘resilient’ (solid red line; N* *=* *111/218; 51%) groups at 0–3, 7 and 30 days after the onset of the child's FN episode. The solid black line depicts Australian population norms (mean = 0.82, SD = not reported)*[17]*. The dotted blue and red lines depict the predicted trajectory of the parent's AQol-8D scores in the ‘chronic and ‘resilient’ groups, respectively. The dotted gray lines depict the 95% confidence intervals of each trajectory group.* Abbreviations: AQoL-8D = Assessment of Quality of Life – 8 Dimensions, FN = febrile neutropenia, HRQoL = health-related quality of life; SD = standard deviation.

For parents, the most optimal model identified two likely trajectories: chronic (*N* = 107/218, 49%) and resilient (*N* = 111/218, 51%) (see Fig. 2). Parents in the chronic group had HRQoL scores that were significantly below Australian population norms [17] at the onset of FN (mean = 0.49; 95% CI: 0.46, 0.53), which did not improve throughout follow-up (mean = 0.51; 95% CI: 0.48, 0.55). Parents in the resilient group had relatively high HRQoL scores, that were marginally below population norms at the onset of FN (mean = 0.77; 95% CI: 0.74, 0.80) and improved to slightly above Australian population norms after the resolution of the episode (mean = 0.82; 95% CI: 0.80, 0.85). Consistent with the results of the child trajectory model, parents in the chronic group appeared to be unaffected by the child's FN episode as their HRQoL scores remained unchanged after the resolution of the FN episode (MD = 0.04; 95% CI: −0.00, 0.09; p-value > 5%), whilst parents in the resilient group experienced significant gains in HRQoL (MD = 0.05, 95% CI: 0.01, 0.09; p-value ≤ 5%) (see Table S4).

Of the children and parents that had HRQoL information available for more than one FN episode, trajectory group membership remained relatively stable between FN episodes. Results from transition matrices indicate that 64% (*N* = 32/50) of children and 85% (*N* = 57/73) of parents who experienced more than one FN episode were reassigned to the same HRQoL trajectory group for those subsequent episodes (see Table S5). Of the participants who transitioned to a group different to their initial group (*N* = 11/18; 61% of children and *N* = 5/11; 45% of parents), it was more common for children to be assigned to a trajectory group that suggested the participant was experiencing a worsening in HRQoL trajectory (i.e. movement from recovery to chronic or resilient to chronic or recovery) with multiple episodes. For parents, there was no clear pattern in movement between groups over multiple episodes.

### 3.3 Risk factors for trajectory group membership

Results of multivariate analyses found that the child being male (relative risk (RR) for child = 6.26; 95% CI: 1.49, 25.00; and RR for parent = 3.33; 95% CI: 1.28, 9.09) having solid cancer (RR for child = 11.36; 95% CI: 1.99, 64.87; and RR for parent = 3.6; 95% CI: 1.33, 9.77), undergoing intensive chemotherapy (RR for child =10.40; 95% CI: 1.78, 60.98; and RR for parent = 7.49; 95% CI: 1.48, 38.02), parents experiencing financial stress (RR for child =10.54; 95% CI: 2.39, 46.42; and RR for parent = 9.51; 95% CI: 3.27, 27.68) and the presence of relationship difficulties between parent and child (RR for child = 4.23; 95% CI: 1.30, 13.73; and RR for parent = 8.95; 95% CI: 2.42, 33.08), significantly increased the odds of chronic group membership relative to being in the resilient group for both parents and children (see Table 2 and Table S7). Children who were classified as high-risk during the FN episode had increased odds of being classified in the recovery group relative to the resilient group (RR = 5.27; 95% CI: 1.06, 26.32). These exposure factors remained significant (p-value < 5%) when using logistic and multinomial regression with cluster robust standard errors to account for serial correlation (Table S7). The child's age and time from cancer diagnosis at the onset of FN had no significant impact (p-value ≥ 5%) on group membership for parents or children (Table 2 and Table S7).

**Table 2:** Predictors of group trajectory group assignment using risk-factor analysis (reference group = resilient). Results are reported as odds ratio (95% confidence intervals).

Risk Factor	Child		Parents
	Chronic vs. Resilient	Recovering vs. Resilient	Chronic vs. Resilient
**Demographics**			
Child is female	0.49 (0.22, 0.99)*	0.51 (0.17, 1.53)	0.03 (0.16, 0.85)**
Mother completed the HRQoL survey	NE	NE	0.97 (0.11, 8.95)
Parent completing the HRQoL survey is university educated	0.94 (0.41, 2.17)	40 (0.40, 3.73)	1.27 (0.24, 6.82)
Child's age	1.01 (0.92, 1.11)	1.06 (0.92, 1.22)	0.99 (0.93, 1.06)
Child completed HRQoL survey	0.96 (0.43, 2.14)	1.13 (0.4, 3.19)	1.00 (0.51, 1.98)
**Quality and type care received during the FN Episode**			
Time spent in ED (hrs)	1.01 (0.94, 1.09)	0.96 (0.86, 1.08)	1.05 (0.99, 1.12)
Time to first antibiotic after hospital presentation (hrs)	1.64 (0.83, 3.23)	1.69 (0.75, 3.81)	1.02 (0.71, 1.46)
Treated at home (HITH)	1.58 (0.47, 5.26)	0.55 (0.07, 4.01)	3.12 (1.11, 8.74)*
Patient presented at the ED during FN episode	0.56 (0.23, 1.34)	0.62 (0.20, 1.92)	1.22 (0.63, 2.36)
**Severity of the FN episode**			
Length of hospital stay (days)	1.12 (1.00, 1.25)*	1.12 (0.99, 1.27)+	1.00 (0.98, 1.03)
Duration of FN episode (days)	1.06 (0.87, 1.29)	1.00 (0.74, 1.35)	1.04 (0.88, 1.22)
Days between FN onset and previous FN episode	1.00 (0.99, 1.01)	1.00 (0.99, 1.02)	1.01 (1, 1.02)+
Duration of antibiotics (days)	1.08 (1.00, 1.17)*	1.02 (0.92, 1.15)	1.02 (0.98, 1.07)
PICNICC FN risk status – child is at high risk	1.97 (0.81, 4.82)	5.05 (1.01, 26.4)*	1.23 (0.58, 2.59)
**Related to the cancer diagnosis**			
Solid Cancer	2.69 (1.13, 6.43)*	0.4 (0.09, 1.74)	2.53 (1.28, 4.98)**
Days between chemo and FN onset	0.96 (0.89, 1.04)	0.98 (0.89, 1.08)	0.95 (0.89, 1.02)
Time with cancer (months)	0.98 (0.93, 1.03)	1.01 (0.96, 1.05)	0.99 (0.95, 1.03)
Intensive chemotherapy	5.35 (1.8, 15.87)**	2.46 (0.72, 8.46)	3.58 (1.31, 9.79)**
**Family situation**			
I have relationship problems with my child?	5.22 (1.62, 16.79)**	4.9 (1.06, 22.56)*	5.61 (2.62, 12)***
I have financial problems due to my child's cancer?	10.58 (0.32, 350.52) b	1.08 (0.31, 3.68)	4.6 (1.83, 11.56)**
**Multivariate Model**			
Child is female	0.16 (0.04, 0.67)**	0.42 (0.12, 1.5)	0.30 (0.11, 0.78)**
Child's age	0.87 (0.74, 1.04)	0.98 (0.85, 1.14)	1.04 (0.94, 1.14)
Time with cancer (months)	1.03 (0.95, 1.11)	1.00 (0.94, 1.07)	1.03 (0.97, 1.09)
PICNICC FN risk status – child is at high risk	3.20 (0.85, 12.05)+	5.27 (1.06, 26.32)*	2.16 (0.74, 6.35)
Intensive chemotherapy	10.40 (1.78, 60.98)**	3.08 (0.68, 13.99)	7.49 (1.48, 38.02)**
Solid Cancer	11.36 (1.99, 64.87)**	0.98 (0.17, 5.81)	3.6 (1.33, 9.77)**
I have relationship problems with my child?	4.23 (1.30, 13.73)**	2.59 (0.65, 10.31)	8.95 (2.42, 33.08)**
I have financial problems due to my child's cancer?	10.54 (2.39, 46.42)**	1.61 (0.3, 8.67)	9.51 (3.27, 27.68)***

Abbreviations: CarerQol = Care-related Quality of Life instrument, ED = emergency department, FN = febrile neutropenia, HITH = hospital in the home, HRQoL = health-related quality of life, NE = not estimable, PICNICC = Predicting Infectious Complications in Children with Cancer, *p*-value ≤ 10 = +, *p*-value ≤ 0.05 = *, *p*-value ≤ 0.01 = **, *p*-value ≤ 0.001 = ***.

Notes:.

^a^Variable specific analyses are univariate unless otherwise specified.

b Financial stress was negatively correlated with the child having solid cancer. After controlling for cancer type, financial stress was significantly predictive of chronic trajectory group membership for children. Further, financial stress was predictive of chronic membership in univariate analyses when using multinomial logistic regression with cluster robust standard errors (see Table S7).

## 4 Discussion

Our study provides a comprehensive insight into the impact FN has on HRQoL in children with cancer and their parents using validated instruments, which allowed for comparisons between patients in our study and Australian population norms [9,10]. Further, this is the first time the CHU9D has been used to assess HRQoL in a pediatric cancer population. Our results show that the onset of an FN episode had a significant and heterogeneous impact on the HRQoL of children with cancer and their parents. For children and parents in the resilient and recovery trajectory groups, the onset of FN had a temporary deleterious impact on HRQoL, which dissipated following FN resolution. However, for children and parents in the chronic groups, there was no improvement following FN resolution suggesting the low HRQoL may be unrelated to the FN episode. In support of this hypothesis, and consistent across parent and child models, time-invariant factors predictive of chronic group membership were the child being male, the child having solid cancer or undergoing intensive chemotherapy, experiencing financial stress and the presence of relationship difficulties between parent and child.

Group based trajectory modeling was chosen as it simultaneously identifies latent (unobserved) homogenous subgroups of individuals with similar trajectories and describes the course of HRQoL within those subgroups [18,19]. This allowed us to identify subpopulations of children and parents with chronically low HRQoL scores, which were significantly below population norms [9,10], at both the onset of an FN episode and up to 30-days after. We were also able to identify children and parents with HRQoL scores that quickly recovered following the resolution of the FN episode (i.e. ‘resilient’ and ‘recovery’ groups). In understanding the existence of such trajectory groups and the characteristics which differentiated them, it may be possible to identify ‘at risk’ subpopulations with early divergent trajectories as potential intervention targets [11].

Using GBTM we were able to identify factors that were associated with children and parents quickly recovering from poor HRQoL following the onset of an FN episode. We found that being female, or being the parent of a female child, was significantly associated with increased odds of being in the resilient group relative to the chronic group for both children and parents. Linear regressions (Table S6) found that being female was a protective factor for HRQoL in acute care situations, even after adjusting for various demographic and clinical factors. This result contradicts prior research in pediatric cancer patients which suggest that being female is associated with poorer HRQoL both during active anti-cancer treatment [20] and up to five years post-treatment [21,22].

The association of solid cancers and intensive chemotherapy regimens with poorer and chronic HRQoL was consistent with previous research in pediatric oncology [20,23]. Intensive chemotherapy regimens require more frequent hospital visits and hospitalization and are associated with more side effects such as mucositis, pain or infective episodes. Furthermore, the prognosis of many solid tumours is worse than for ALL [24]. A strong association between financial distress and reduced HRQoL in both children and their parents was also evident. Moreover, 80% of parents reported financial stress due to their child's cancer. This was despite Australia having a dedicated income support payment for carers of children with chronic illness as well as additional support available from some cancer charities [25]. Previous research has found a strong correlation between financial stress and reduced HRQoL in adult cancer patients [26]. Our results suggest that this impact extends to pediatric cancer patients. Families reporting financial distress have higher odds of experiencing worse HRQoL that persists despite the resolution of the FN episode. These are important finding given that a validated tool exists to identify those experiencing financial distress [27]. Not surprisingly, we also found that families in which there is some degree of relationship distress between parents and children were significantly more likely to be assigned to the chronic group. Identifying families experiencing financial or relationship stress at the commencement of cancer treatment may enable targeted interventions to support both the child and parents throughout the treatment course.

Safely reducing exposure to antibiotics and early hospital discharge, through the use of risk-stratification, have been proposed as ways to improve children's HRQoL in the setting of FN care, while minimizing disruption to family life [2]. This has led to the development of important treatment pathways, focusing on the early discharge to home-based care of patients who are at low risk for serious infection [7]. In keeping with this finding, our results did find a significant association between increased LOS and antibiotic duration, and increased odds of chronic group membership. In contrast, results of our trajectory modeling did not find an association between receipt of home-based care via Hospital-in-the-home programs and HRQoL, for either parents or children (Table 2 and Table S7). Of note, formal low-risk FN programs were not implemented during this study, and the home-based care was likely due to other reasons such as treatment of established infections or other cancer-related complications. Discrete choice experiments that have examined treatment preferences of parents with children undergoing active cancer treatment have found that many parents still prefer inpatient management over outpatient management for low-risk FN episodes [4,6], whilst parents who valued HRQoL were more likely to opt for outpatient care [4]. More recently, a clear preference for home-based treatment of low-risk FN was identified by clinicians and parents due to perceived improvements in HRQoL [28]. This highlights the importance of consumer engagement when home-based FN care pathways are developed and implemented to ensure that parent and patient concerns and reservations are appropriately addressed.

A 2016 systematic review examining early versus later discharge of pediatric children with FN, found no studies reported HRQoL [29]. A randomised controlled trial, comparing inpatient and outpatient treatment of pediatric FN that was not included in this review, did show that outpatient management of low-risk FN provided significant benefit to parents and patients across several HRQoL domains [5]. Our study is, however, the largest study of its kind and provides a comprehensive baseline understanding of the HRQoL impact of standard inpatient management of FN. It is also the first to quantify pediatric and parent HRQoL associated with FN using the validated CHU9D and AQoL-8D tools, and to do so in a manner that can be directly incorporated into economic evaluations. We present results for HRQoL over an acute period (0–7 days after FN onset) and after the resolution of the FN episode (7–30 days after FN onset). This 30-day follow-up period reflects the expected period for resolution and recovery from an FN event [16]. However, as we are the first study to examine the HRQoL of children with cancer using the CHU9D, it was not possible to determine whether children in our study fully recovered from their FN episode at 30-days follow-up by benchmarking against other CHU9D results from other studies in which children were not experiencing FN. Further, as our study only captured information on cancer treatment prior to the FN event but not during 30-day follow-up period, it is unknown if children had recommenced chemotherapy. This is particularly important for children with solid cancers who typically receive cancer treatments in 21-day cycles.

Febrile neutropenia has a significant and heterogeneous impact on the HRQoL of children with cancer and their parents in resilient and recovery trajectory groups. However, for children and parents in the chronic group, their persistently low HRQoL remained static throughout. Risk factors for chronic group members include the child being male, having solid cancer, undergoing intensive chemotherapy, financial stress and the presence of relationship difficulties between parent and child. These factors could be used to identify children and families who would benefit from additional financial and psychological support during their cancer care. Combined with our improved understanding of pediatric FN, our results provide critical insights for the current and future management of pediatric cancer patients and their families.

## Funding

This study was funded by a National Health and Medical Research Council Grant (APP1104527).

## Author contribution

All authors were involved in study design and had full access to all the data in the study. Gabrielle M Haeusler, Francoise Mechinaud, Heather Tapp, Bhavna Padhye, David Zeigler, Frank Alvaro, Leanne Super, Thomas Walwyn and Julia Clark oversaw data collection. Anna Crothers and Richard De Abreu Lourenco performed the analysis and all authors provided clinical interpretation of the findings. Anna Crothers, Gabrielle M Haeusler and Richard De Abreu Lourenco drafted the manuscript. All authors reviewed, edited and confirmed their acceptance of the final submitted version.

## Data sharing statement

The datasets generated during and analysed during this study are available from the corresponding author (Anna Crothers), on reasonable request.

## Declaration of Competing Interest

DZ reports personal fees from Bayer, personal fees from Amgen and personal fees from Day One, outside the submitted work. AC reports grants from NHMRC APP1104527, during the conduct of the study. GMH reports grants from NHMRC APP1104527 and the Victorian Cancer Agency early career fellowship, during the conduct of the study. MAS reports grants from Merck, from Gilead Sciences, and from F2G, personal fees from Pfizer and personal fees from Gilead Sciences, outside the submitted work. RDAL reports grants from NHMRC APP1104527, during the conduct of the study.  RP reports grants from NIHR, during the conduct of the study. All the other authors report no conflicts.

## Appendix Supplementary materials

Image, application 1
