# Supplementary material for: Examining health-related quality of life in pediatric cancer patients with febrile neutropenia: Factors predicting poor recovery in children and their parents
Source: eClinicalMedicine. 2021 Aug 20;40:101095. doi: 10.1016/j.eclinm.2021.101095 (PMC8548915; doi:10.1016/j.eclinm.2021.101095)
Supplement: Supplementary file 1 [file mmc1.docx]

# Online Supplementary Material

## Additional analyses

**Table S3: Comparison of demographics for FN episodes with available HRQoL data compared to FN episodes without available HRQoL data**

| **Mean (standard deviation), unless otherwise specified** | **Sample** | **Missing** |
| --- | --- | --- |
| **Total number of FN episodes** | **234** | **624** |
| Number of FN episodes per patient with available HRQoL data *  - One FN episode  - Two FN episodes  - Three FN episodes  - ≥ Four FN episodes | 105 (45%)  90 (39%)  18 (8%)  21 (9%) | 179 (29%)  173 (28%)  129 (21%)  143 (23%) |
| Mean number of FN episodes reported per child with available HRQoL data | 12.53 (1.34) | 2.7 (1.8) |
| Mean number of days between FN episodes | 25.9 (40.2) | 23.8 (40.0) |
| Patients was transferred to:  - Hospital care  - Home care (HITH) | 209 (89%)  26 (11%) | 550 (88%)  74 (12%) |
| Child’s sex  - Male  - Female | 115 (49%)  119 (51%) | 328 (53%)  296 (47%) |
| Mean child’s age at FN onset (years) | 7.8 (4.8) | 7.17 (4.9) |
| Cancer Type *  - Blood cancer (lymphoma/leukemia)  - Solid cancer | 155 (66%)  79 (34%) | 360 (58%)  264 (42%) |
| Mean length of hospital stay (days) * | 8.5 (11.5) | 10.43 (15.60) |
| Length of hospital stay (dichotomised at median) *  - 0-5 days  - > 5 days | 121 (52%)  113 (48%) | 284 (46%)  340 (54%) |
| Chemotherapy intensity  - Non-intensive  - Intensive | 35 (15%)  199 (85%) | 65 (10%)  559 (90%) |
| Mean duration of antibiotic treatment (days) | 7.3 (7.8) | 8.5 (10.0) |
| Duration of antibiotic treatment (dichotomised at median)  - 0-4 days  - > 4 days | 104 (44%)  130 (56%) | 252 (40%)  372 (60%) |
| Mean duration of FN episode (days) | 1.3 (2.1) | 1.7 (2.8) |
| Mean time to first antibiotic after hospital presentation, hours (SD) | 1.0 (0.8) | 1.0 (0.0) |
| Time to first antibiotic  - Within 1 hour after hospital presentation  - > 1 hour after hospital presentation | 142 (60%)  92 (39%) | 413 (66%)  211 (34%) |
| Mean time with cancer (months) | 7.9 (9.9) | 7.8 (11.1) |
| Time with cancer (dichotomised at median)  - ≤ 4.5 months  - > 4.5 months | 113 (48%)  121 (52%) | 310 (50%)  314 (50%) |
| PICNICC FN risk status  - Low risk  - High risk | 46 (20%)  188 (80%) | 109 (17%)  515 (83%) |

Abbreviations: AQoL-8D = Assessment of Quality of Life – 8 Dimensions, CarerQol = Care-related Quality of Life instrument, CHU9D = CHU9D – 9 Dimensions, CI = confidence intervals, ED = emergency department, FN = febrile neutropenia, HITH = hospital in the home, HRQoL = health-related quality of life, NE = not estimable, PICNICC = Predicting Infectious Complications in Children with Cancer, * = denotes statistical significance at p-value ≤ 0.05

**Table S4: Mean utility values (95% confidence intervals) at each time point by group membership**

| **Mean utility values (95% CI)** | **Overall** | **Chronic** | **Recovering** | **Resilient** |
| --- | --- | --- | --- | --- |
| **Parents (AQoL-8D)** | | | | |
| 0-1-day post FN onset | N = 152,  0.65 (0.62, 0.68) | N = 66  0.49 (0.46, 0.53) | - | N = 86  0.77 (0.74, 0.80) |
| 7-days post FN onset | N = 150  0.65 (0.62, 0.68) | N = 72  0.48 (0.46, 0.51) | - | N = 78  0.80 (0.77, 0.82) |
| 30-days post FN onset | N = 138  0.68 (0.62, 0.71) | N = 66  0.51 (0.48, 0.55) | - | N = 72  0.82 (0.80, 0.85) |
| Mean change at 7-days post FN onset from 0-3 days post FN onset | N = 103  0.02 (-0.01, 0.04) | N = 41  -0.01 (-0.05, 0.03) | - | N = 62  0.04 (0.01, 0.06) * |
| Mean change at 30-days post FN onset from 0-3 days post FN onset | N = 93  0.05 (0.02, 0.08) ** | N = 36  0.04 (-0.00, 0.09) | - | N = 57  0.05 (0.01, 0.09) * |
| Mean change at 30-days post FN onset from 7 days post FN onset | N =103  0.02 (-0.00, 0.05) | N = 49  0.04 (-0.00, 0.07) | - | N = 54  0.01 (-0.02, 0.04) |
| **Children (CHU9D)** | | | | |
| 0-1-day post FN onset | N = 120  0.39 (0.34, 0.44) | N = 51  0.19 (0.14, 0.23) | N = 23  0.25 (0.19, 0.30) | N = 46  0.68 (0.64, 0.72) |
| 7-days post FN onset | N = 117  0.48 (0.43, 0.54) | N = 50  0.20 (0.17, 0.23) | N = 29  0.54 (0.49, 0.60) | N = 38  0.81 (0.75, 0.86) |
| 30-days post FN onset | N = 96  0.51 (0.45, 0.58) | N = 40  0.21 (0.17, 0.26) | N = 24  0.58 (0.49, 0.67) | N = 32  0.84 (0.79, 0.89) |
| Mean change at 7-days post FN onset from 0-3 days post FN onset | N = 82  0.15 (0.10, 0.20) *** | N = 28  0.08 (-0.06, 0.08) | N = 21  0.34 (0.27, 0.41) *** | N = 33  0.15 (0.07, 0.23) *** |
| Mean change at 30-days post FN onset from 0-3 days post FN onset | N = 68  0.15 (0.08, 0.21) *** | N = 26  0.02 (-0.07, 0.11) | N = 16  0.30 (0.14, 0.46) ** | N = 26  0.17 (0.09, 26) *** |
| Mean change at 30-days post FN onset from 7 days post FN onset | N = 71  0.02 (-0.04, 0.08) | N = 27  0.00 (-0.6, 0.06) | N = 17  0.03 (-0.16, 0.23) | N = 27  0.04 (-0.05, 0.14) |

Abbreviations: AQoL-8D = Assessment of Quality of Life – 8 Dimensions, CHU9D = CHU9D – 9 Dimensions, CI = confidence intervals, FN = febrile neutropenia, N = number, p-value ≤ 10 = +, p-value ≤ 0.05 = *, p-value ≤ 0.01 = **, p-value ≤ 0.001 = ***

**Table S5: Transition matrix of child and parent’s prior and subsequent trajectory group assignment**

| **Prior** | | **Subsequent** | | | | | | | | | | |
| --- | --- | --- | --- | --- | --- | --- | --- | --- | --- | --- | --- | --- |
|  |  | **Chronic** | | **Recovering** | | **Resilient** | | **Stable** | | **Worsening** | | **Improving** |
| **Child** | | | | | | | | | | | | |
| Chronic | 21 (75%) | | 3 (30%) | | 4 (33%) | | 21 (66%) | | 0 | | 7 (100%) | |
| Recovering | 5 (18%) | | 3 (30%) | | 0 | | 3 (10%) | | 5 (45%) | | 0 | |
| Resilient | 2 (7%) | | 4 (40%) | | 8 (67%) | | 8 (25%) | | 6 (55%) | | 0 | |
| **Parent** | | | | | | | | | | | | |
| Chronic | 34 (87%) | | - | | 6 (21%) | | 57 (87%) | | 0 | | 6 (100%) | |
| Resilient | 5 (13%) | | - | | 23 (79%) | | 5 (13%) | | 5 (100%) | | 0 | |

Abbreviations: Stable = ‘Chronic to Chronic’ OR ‘Recovering to Recovering’ OR ‘Resilient to Resilient’ ; Worsening = ‘Resilient to Chronic / Recovering’ OR ‘Recovering to Chronic’; Improving = ‘Recovering to Resilient’ OR ‘Chronic to Recovering / Resilient’

**Table S6: Analyses of child and parent’s HRQoL at timepoint: 0-1, 7 and 30 days after on the onset the child’s FN episode**

| **Population** | **Child HRQoL (CHU9D)** | | | **Parent HRQoL (AQol-8D)** | | |
| --- | --- | --- | --- | --- | --- | --- |
| **Time since FN onset** | **0-3 days** | **7-days** | **30-days** | **0-3 days** | **7-days** | **30-days** |
| **Demographics** | | | | | | |
| Child is female | 0.177*** | 0.113 | 0.029 | 0.100** | 0.067 | 0.049 |
| Mother completed the HRQoL survey | 0.156 | 0.111 | -0.044 | 0.056 | 0.193** | -0.075 |
| Parent completing the HRQoL survey is university educated | -0.003 | 0.015 | 0.043 | -0.031 | -0.021 | 0.01 |
| Child's age | 0.00 | -0.011 | -0.019* | 0.007* | 0.00 | 0.001 |
| Child completed HRQoL survey | -0.014 | 0.069 | 0.09 | 0.004 | 0.017 | 0.029 |
| **Symptoms of the FN episode** | | | | | | |
| Number of FN episodes reported | -0.013 | -0.022 | -0.036 | -0.015 | -0.014 | 0.019 |
| Max temperature | 0.006 | 0.031 | -0.035 | -0.046** | -0.023 | -0.01 |
| Sepsis | 0.05 | 0.076 | 0.01 | -0.048* | -0.005 | -0.011 |
| Rigors | -0.093 | -0.018 | -0.054 | -0.008 | 0.035 | 0.019 |
| Vomiting | -0.095 | -0.094 | -0.085 | -0.027 | 0.001 | -0.002 |
| Hypotension | -0.022 | -0.062 | -0.249* | 0.021 | -0.044 | -0.095* |
| Mucositis (moderate to severe) | -0.156* | -0.067 | -0.089 | 0.071* | 0.01 | 0.023 |
| Respiratory effort | -0.118 | -0.118 | 0.053 | 0.071 | -0.042 | 0.013 |
| Tachycardia | 0.063 | -0.025 | 0.079 | -0.032 | 0.011 | 0.015 |
| Tachypnoea | 0.52* | NE | 0.169 | -0.281* | NE | -0.184* |
| **Quality and type care received during the FN Episode** | | | | | | |
| Time spent in ED (hrs) | 0.004 | -0.001 | 0.004 | -0.002 | 0.000 | 0.000 |
| Time to first antibiotic after hospital presentation (hrs) | -0.031 | -0.056 | -0.023 | 0.009 | -0.005 | -0.010 |
| Patient transferred to homecare (HITH) | -0.109 | -0.139 | -0.035 | -0.029 | -0.005 | -0.101* |
| Patient presented at the ED during FN episode | 0.086 | 0.037 | 0.060 | -0.035 | 0.011 | 0.032 |
| **Severity of the FN episode** | | | | | | |
| Length of hospital stay (days) ^b^ | -0.004* | -0.011** | 0.001 | -0.001 | -0.001 | 0 |
| Duration of FN episode (days) | -0.008 | -0.008 | 0.001 | -0.002 | -0.007 | 0.015* |
| Time between FN episodes (days) | 0.000 | -0.002** | 0.000 | 0.000 | 0.000 | 0.000 |
| Duration of antibiotics (days) ^b^ | -0.010*** | -0.009* | 0.000 | -0.003 | -0.001 | -0.004* |
| PICNIC CDR - high risk | -0.083 | -0.135* | -0.04 | -0.053 | -0.05 | -0.033 |
| **Related to the cancer diagnosis** | | | | | | |
| Solid Cancer | -0.069 | -0.121 | -0.242** | -0.02 | -0.056 | -0.082* |
| Days between chemo and FN onset | 0.006 | 0.008 | 0.007 | 0.002 | 0.007** | 0.004 |
| Time with cancer (months) | 0.001 | 0.002 | 0.002 | -0.001 | 0.001 | 0.001 |
| Intensive chemotherapy | -0.148* | -0.157* | -0.215** | -0.026 | -0.06 | -0.114** |
| **Psychosocial** | | | | | | |
| I have relationship problems with my child? | -0.224*** | -0.131* | -0.123 | -0.101*** | -0.105*** | -0.112*** |
| I have financial problems due to my child’s cancer? | -0.099 | -0.206** | -0.312*** | -0.131** | -0.102** | -0.120** |
| **Multivariate Model** | | | | | | |
| Child is female | 0.148** | 0.100 | 0.094 | 0.098** | 0.062 | 0.065 |
| Child's age | 0.003 | -0.001 | -0.003 | 0.005 | 0.000 | 0.000 |
| Time with cancer (months) | -0.002 | -0.001 | -0.001 | -0.001 | 0.000 | 0.000 |
| PICNICC FN risk status - high risk | -0.106* | -0.141* | -0.052 | -0.052 | -0.047 | -0.020 |
| Intensive chemotherapy | -0.102 | -0.097 | -0.178* | -0.007 | -0.045 | -0.072 |
| Solid Cancer | -0.125* | -0.156* | -0.244*** | -0.045 | -0.060 | -0.082* |
| I have relationship problems with my child? | -0.202*** | -0.103 | -0.162** | -0.118** | -0.070 | -0.111** |
| I have financial problems due to my child’s cancer? | -0.086 | -0.162* | -0.315*** | -0.075** | -0.100*** | -0.098** |

Abbreviations: AQoL-8D = Assessment of Quality of Life – 8 Dimensions, CarerQol = Care-related Quality of Life instrument, CHU9D = CHU9D – 9 Dimensions, CI = confidence intervals, ED = emergency department, FN = febrile neutropenia, HITH = hospital in the home, HRQoL = health-related quality of life, NE = not estimable, PICNICC = Predicting Infectious Complications in Children with Cancer, p-value ≤ 10 = +, p-value ≤ 0.05 = *, p-value ≤ 0.01 = **, p-value ≤ 0.001 = ***

Notes:

^a^ Variable specific analyses are univariate unless otherwise specified.

^b^ Duration of antibiotic treatment and length of hospital stay is significantly correlated with FN risk status. Hence, these variables were excluded from multivariate analyses.

**Table S7: Predictors of group trajectory group assignment using multinomial logistic regression (cluster robust standard errors = child ID). Results are reported as relative risk ratio (95% confidence intervals)**

| **Risk Factor** | **Child** | | **Parents** |
| --- | --- | --- | --- |
|  | **Chronic vs. Resilient** | **Recovering vs. Resilient** | **Chronic vs. Resilient** |
| **Demographics** | | | |
| Child is female | 0.46 (0.2, 1.08) + | 0.66 (0.26, 1.7) | 0.48 (0.24, 0.95)** |
| Mother completed the HRQoL survey | NE | NE | 0.94 (0.15, 5.80) |
| Parent completing the HRQoL survey is university educated | 0.92 (0.44, 1.94) | 1.21 (0.47, 3.1) | 0.60 (0.18, 2.04) |
| Child's age | 1.02 (0.92, 1.14) | 0.91 (0.81, 1.02) | 1.00 (0.93, 1.07) |
| Child completed HRQoL survey | 0.96 (0.43, 2.11) | 1.12 (0.47, 2.68) | 0.92 (0.43, 1.96) |
| **Quality and type care received during the FN Episode** | | | |
| Time spent in ED (hrs) | 1.05 (0.95, 1.15) | 0.98 (0.88, 1.1) | 1.06 (1, 1.12)+ |
| Time to first antibiotic after presenting at the hospital (hrs) | 1.65 (0.94, 2.88)+ | 1.4 (0.72, 2.71) | 3.53 (0, 20.9 x 10^8^) |
| Patient transferred to homecare (HITH) | 1.60 (0.52, 4.89) | 0.55 (0.09, 3.2) | 2.77 (1.13, 6.83)** |
| Patient presented at the ED during FN episode | 0.52 (0.23, 1.18) | 0.58 (0.23, 1.43) | 1.37 (0.75, 2.5) |
| **Severity of the FN episode** | | | |
| Length of hospital stay (days) | 1.14 (1.06, 1.23)** | 1.12 (1.04, 1.22)** | 1 (0.97, 1.02) |
| Duration of FN episode (days) | 1.6 (0.76, 3.37) | 2.06 (0.93, 4.57)+ | 1.04 (0.9, 1.2) |
| Days between FN onset and previous FN episode | 1.01 (0.23, 1) | 1.01 (1, 1.02) | 1.01 (1, 1.02)** |
| Duration of antibiotics (days) | 1.1 (1.03, 1.16)** | 0.97 (0.87, 1.08) | 1.02 (0.98, 1.06) |
| PICNICC FN risk status – child is at high risk | 2.13 (0.86, 5.29) | 4.13 (1.21, 14.02)* | 1.32 (0.62, 2.81) |
| **Related to the cancer diagnosis** | | | |
| Solid Cancer | 3.33 (1.27, 8.69)* | 0.33 (0.1, 1.05)+ | 2.53 (1.15, 5.57)** |
| Days between chemo and FN onset | 0.97 (0.9, 1.04) | 1 (0.92, 1.09) | 0.96 (0.91, 1.01) |
| Time with cancer (months) | 0.97 (0.93, 1.02) | 1.01 (0.98, 1.04) | 0.99 (0.97, 1.02) |
| Intensive chemotherapy | 5.07 (1.91, 13.4)** | 1.94 (0.74, 5.12) | 3.04 (1.3, 7.09)** |
| **Family Situation** | | | |
| I have relationship problems with my child? | 4.2 (1.74, 10.13)** | 3.25 (1.35, 7.82)** | 5.39 (2.71, 10.7)*** |
| I have financial problems due to my child’s cancer? | 4.57 (1.94, 10.74)*** | 0.98 (0.42, 2.29) | 4.62 (1.72, 12.45)** |
| Parent’s trajectory is chronic (vs. resilient) | 14.36 (4.98, 41.39)*** | 2.44 (0.95, 6.27)+ | - |
| Child’s trajectory group is chronic (vs. resilient) | - | - | 15.71 (5.24, 47.13)*** |
| Child’s trajectory group is recovering (vs. resilient) | - | - | 4.06 (1.06, 15.61)** |
| **Multivariate Model** | | | |
| Child is female | 0.07 (0.01, 0.56)* | 0.40 (0.14, 1.15)+ | 0.24 (0.09, 0.62)** |
| Child's age | 0.79 (0.64, 0.96)* | 0.96 (0.85, 1.08) | 1.06 (0.96, 1.17) |
| Time with cancer (months) | 1.07 (0.99, 1.15) | 1.00 (0.95, 1.06) | 1.05 (1.01, 1.1)** |
| PICNICC FN risk status – high | 1.29 (0.25, 6.58) | 6.95 (1.93, 24.99)** | 2.08 (0.81, 5.36) |
| Intensive chemotherapy | 73.67 (5.14, 1056)** | 3.72 (1.07, 12.92)* | 10.67 (2.87, 39.75)*** |
| Solid Cancer | 516.85 (22.61, 11813.32)*** | 1.54 (0.36, 6.47) | 3.86 (1.47, 10.16)** |
| I have relationship problems with my child? | 29.99 (6.44, 139.67)*** | 3.08 (1.13, 8.43)* | 13.26 (2.29, 76.75)** |
| I have financial problems due to my child’s cancer? | 17.34 (2.63, 114.47)** | 1.76 (0.63, 4.92) | 10.01 (4.05, 24.73)*** |

Abbreviations: CarerQol = Care-related Quality of Life instrument, ED = emergency department, FN = febrile neutropenia, HITH = hospital in the home, HRQoL = health-related quality of life, NE = not estimable, PICNICC = Predicting Infectious Complications in Children with Cancer, p-value ≤ 10 = +, p-value ≤ 0.05 = *, p-value ≤ 0.01 = **, p-value ≤ 0.001 = ***

Note:

^a^ Variable specific analyses are univariate unless otherwise specified.

## Model Fit

**Table S8: Model fit for the child population**

| **Groups** | **BIC** | **Chronic** | | | | **Resilient** | | | | **Recovering** | | | | | **Relapsing** | | | |
| --- | --- | --- | --- | --- | --- | --- | --- | --- | --- | --- | --- | --- | --- | --- | --- | --- | --- | --- |
|  |  | **N** | **%** | **APP** | **OCC** | **N** | **%** | **APP** | **OCC** | **N** | **%** | **APP** | **OCC** | **N** | | **%** | **APP** | **OCC** |
| **No covariates** | | | | | | | | | | | | | | | | | | |
| 2 | -50.79 | 108 | 65% | 0.93 | 8.33 | 59 | 35% | 0.94 | 27.73 |  |  |  |  |  | |  |  |  |
| 3 | -44.72 | 78 | 47% | 0.85 | 7.34 | 53 | 32% | 0.92 | 26.83 | 59 | 35% | 0.92 | 26.83 |  | |  |  |  |
| 4 | -43.49 | 72 | 43% | 0.82 | 7.27 | 53 | 32% | 0.92 | 24.41 | 12 | 7% | 0.88 | 48.29 | 30 | | 18% | 0.69 | 9.73 |
| **Child’s age and sex** | | | | | | | | | | | | | | | | | | |
| 2 | -53.49 | 106 | 63% | 0.94 | 9.47 | 61 | 37% | 0.93 | 21.28 |  |  |  |  |  | |  |  |  |
| 3 | -50.96 | 86 | 51% | 0.90 | 9.02 | 59 | 35% | 0.81 | 23.59 | 22 | 13% | 0.81 | 23.59 |  | |  |  |  |
| 4 | -53.53 | 62 | 37% | 0.82 | 7.97 | 53 | 32% | 0.93 | 29.73 | 18 | 11% | 0.75 | 21.01 | 29 | | 17% | 0.75 | 12.15 |

Abbreviations: APP = average posterior probabilities, BIC = Bayesian information criterion, Chronic = persistently low HRQoL scores over the 30-day course of follow-up; HRQoL = health-related quality of life; OCC = odds of correct; Recovering = initially low HRQL scores, which improve over the 30-day course of follow-up; Relapsing = initially low HRQoL score, which improve at 7-days after FN-onset and then decrease at 30-days post-FN onset; Resilient = persistently high HRQoL scores over the 30-day course of follow-up

**Table S9: Model fit for the parent population.**

| **Groups** | **BIC** | **Chronic** | | | | **Resilient** | | | | **Moderate** | | | |
| --- | --- | --- | --- | --- | --- | --- | --- | --- | --- | --- | --- | --- | --- |
|  |  | **N** | **%** | **APP** | **OCC** | **N** | **%** | **APP** | **OCC** | **N** | **%** | **APP** | **OCC** |
| **No covariates** | | | | | | | | | | | | | |
| 2 | 145.69 | 107 | 49% | 0.91 | 10.22 | 111 | 51% | 0.92 | 10.72 |  |  |  |  |
| 3 | 191.54 | 45 | 21% | 0.90 | 33.44 | 76 | 35% | 0.94 | 28.28 | 97 | 44% | 0.94 | 28.28 |
| **Child’s age and sex** | | | | | | | | | | | | | |
| 2 | 143.63 | 104 | 48% | 0.91 | 10.49 | 114 | 52% | 0.92 | 9.84 |  |  |  |  |
| 3 | 186.32 | 43 | 20% | 0.91 | 39.67 | 78 | 36% | 0.93 | 24.16 | 97 | 44% | 0.90 | 10.98 |

Abbreviations: APP = average posterior probabilities, BIC = Bayesian information criterion, Chronic = persistently low HRQoL scores over the 30-day course of follow-up; HRQoL = health-related quality of life; Moderate = moderately high HRQoL scores over the 30-day course of follow-up; OCC = odds of correct; Resilient = persistently high HRQoL scores over the 30-day course of follow-up
